# The trade‐off between investment in weapons and fertility is mediated through spermatogenesis in the leaf‐footed cactus bug *Narnia femorata*

**DOI:** 10.1002/ece3.7686

**Published:** 2021-06-08

**Authors:** Katelyn R. Cavender, Tessa A. Ricker, Mackenzie O. Lyon, Emily A. Shelby, Christine W. Miller, Patricia J. Moore

**Affiliations:** ^1^ Department of Entomology University of Georgia Athens GA USA; ^2^ Entomology and Nematology Department University of Florida Gainesville FL USA

**Keywords:** autotomy, *Narnia femorata*, spermatogenesis, testes, trade‐off, weapons

## Abstract

Males have the ability to compete for fertilizations through both precopulatory and postcopulatory intrasexual competition. Precopulatory competition has selected for large weapons and other adaptations to maximize access to females and mating opportunities, while postcopulatory competition has resulted in ejaculate adaptations to maximize fertilization success. Negative associations between these strategies support the hypothesis that there is a trade‐off between success at pre‐ and postcopulatory mating success. Recently, this trade‐off has been demonstrated with experimental manipulation. Males of the leaf‐footed cactus bug *Narnia* *femorata* use hind limbs as the primary weapon in male–male competition. However, males can drop a hind limb to avoid entrapment. When this autotomy occurs during development, they invest instead in large testes. While evolutionary outcomes of the trade‐offs between pre‐ and postcopulatory strategies have been identified, less work has been done to identify proximate mechanisms by which the trade‐off might occur, perhaps because the systems in which the trade‐offs have been investigated are not ones that have the molecular tools required for exploring mechanism. Here, we applied knowledge from a related model species for which we have developmental knowledge and molecular tools, the milkweed bug *Oncopeltus fasciatus*, to investigate the proximate mechanism by which autotomized *N*. *femorata* males developed larger testes. Autotomized males had evidence of a higher rate of transit amplification divisions in the spermatogonia, which would result more spermatocytes and thus in greater sperm numbers. Identification of mechanisms underlying a trade‐off can help our understanding of the direction and constraints on evolutionary trajectories and thus the evolutionary potential under multiple forms of selection.

## Graphical abstract

Male leaf‐footed cactus bugs that lose the ability to fully invest in weapons required for success in precopulatory contests invest instead in testis size that allows them to compete for postcopulatory fertilization success. In this study, we shed light on the mechanism by which this trade‐off occurs; males that lost their weapon during a key developmental stage have sperm progenitor cells that divided more rapidly than control males. Understanding proximate mechanisms can help us understand potential directions and constraints on the evolution of this trade‐off under different forms of selection.

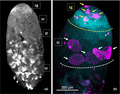

## 1 INTRODUCTION

Male–male competition has led to directional selection on weapon size (Andersson, 1994; Emlen, 2008). Increasing weapon size can result in improved success in competition for territories and mating opportunities, but it can be costly. Extreme weapon size may be countered by the increasing costs through natural selection (O'Brien et al., 2017). This leads to a potential trade‐off between investing in weapons and other important fitness traits, including investment in fertility and other postcopulatory traits (Filice & Long, 2018; Joseph et al., 2018; Lüpold et al., 2014; Somjee et al., 2018). Observational studies have demonstrated the predicted negative association between size of weapons and size of copulatory organs, including testes and ejaculates, supporting the idea of a trade‐off between investment in traits leading to precopulatory and postcopulatory mating success. Males that lack weapons may compensate by investing more in any potential mating opportunity.

The trade‐off between pre‐ and postcopulatory tactics has recently been experimentally tested in the leaf‐footed cactus bug *Narnia* *femorata* Stål (Hemiptera: Coreidae). Males of *N*. *femorata* use their enlarged hind limbs to strike and squeeze other males over access to territories that attract females (Nolen et al., 2017; Procter et al., 2012). While hind limbs are crucial for winning fights with other males (Emberts et al., 2016), twelve percent of adult male *N*. *femorata* in the wild are missing one or more limbs through the process of autotomy (Emberts et al., 2016). Autotomy is used by *N*. *femorata* of all ages to escape entrapment, and autotomy does not reduce their survival in a laboratory setting (Joseph et al., 2018; Miller et al., 2021). Autotomized limbs are not regenerated (Emberts et al., 2017; Emberts et al., 2018). Even though males missing a hind limb are poor competitors with other males, they may still have an opportunity to mate. Females move around a lot, and males may occasionally encounter them alone (C. W. Miller, personal observation). Further, males may avoid other males by sneaking copulations (see Gross, 1996). Indeed, observations of *N*. *femorata* and other leaf‐footed bugs from the wild and in seminatural enclosures have revealed that dominant males patrol territories with multiple females but cannot always keep other males away (C. W. Miller, personal observation).

Experimentally autotomized *N*. *femorata* males that have lost their weapon during development have reduced opportunity to invest in the weapons important for success in precopulatory competition. Instead, autotomized males reallocate resources into postcopulatory reproductive success by growing larger testes (Joseph et al., 2018; Miller et al., 2019; Miller et al., 2021). They also have a fertilization advantage over intact males (Joseph et al., 2018; Cirino et al., 2021). Here, we examine the mechanism of these observed patterns, specifically whether the testes of autotomized male *N*. *femorata* show an increase in sperm production.

While much work has focused on the selection and evolutionary outcome of the trade‐off between pre‐ and postcopulatory success, much less work has been done on the mechanisms by which males might respond to an environment in which that trade‐off occurs. Yet, we know that both genetic and developmental processes can constrain or facilitate the evolution of traits (Smith et al., 1985). Identifying these genetic and developmental mechanisms could be critical to understanding the targets of selection leading to the evolutionary trade‐off (Zera & Harshman, 2001). Mechanisms may be understudied in part because the biological systems in which these trade‐offs are being studied are not easily dissected using modern tools, or their development has not been deeply characterized. *Narnia femorata*, however, belongs to the Hemiptera, a diverse group of bugs that have been widely studied and for which genomic resources are being developed (Panfilio & Angelini, 2018). Here, we take advantage of extensive information on testis development and spermatogenesis in a relative of *N*. *femorata*, the large milkweed bug, *Oncopeltus fasciatus*, to begin to identify the developmental mechanism by which autotomized males may be increasing sperm production that results in larger testis size. While *O*. *fasciatus* does not display autotomy (to our knowledge), other variables have been shown to influence the progress through spermatogenesis (Duxbury et al., 2018).

**FIGURE 1:**
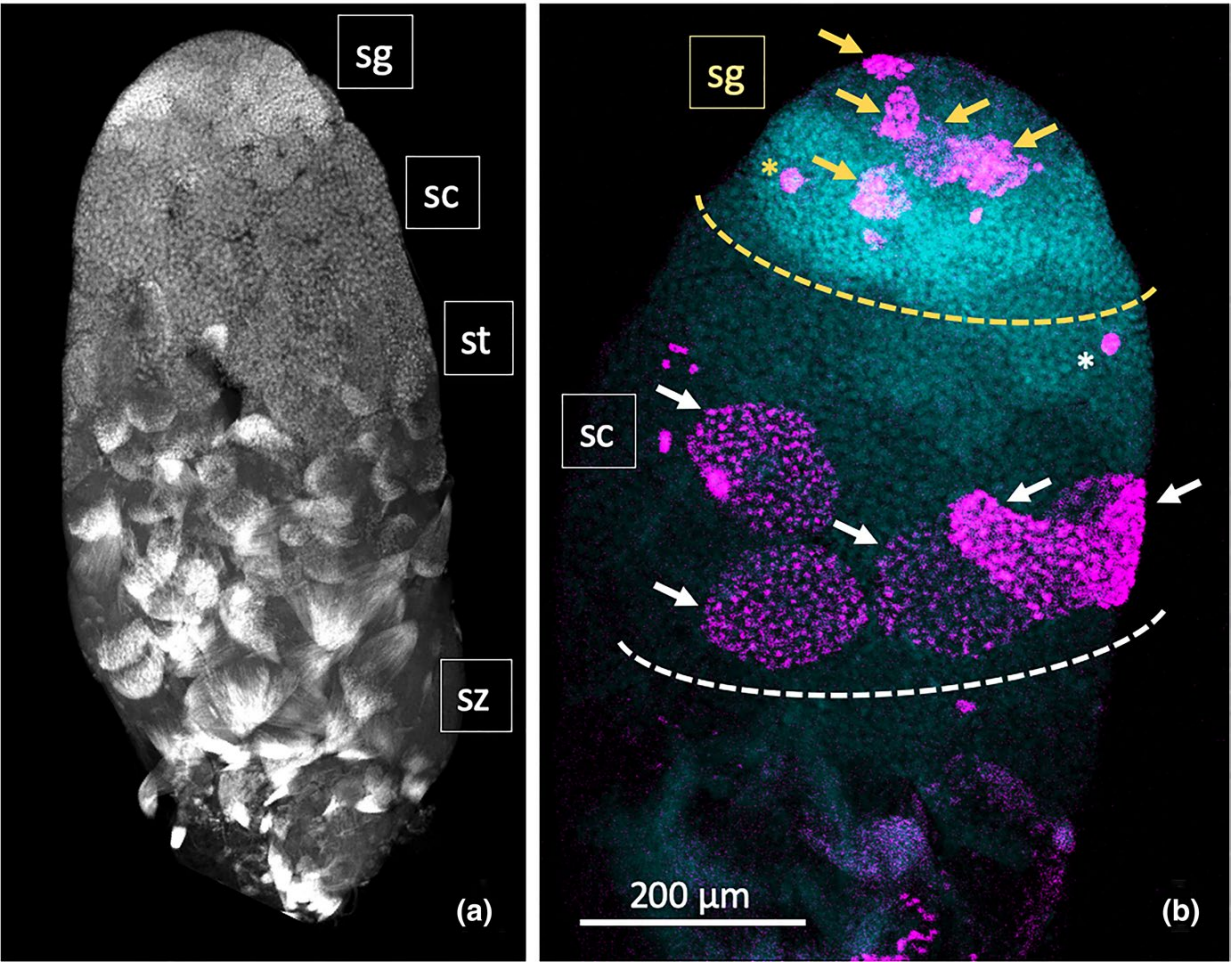
Structure of *Narnia femorata* testis tubule. (a) A low‐magnification image (6×) of a *N*. *femorata* testis tubule stained with DAPI (DNA). The progression through spermatogenesis could be clearly differentiated based on nuclear morphology. At the apical tip, spermatocysts containing spermatogonia were identified based on the number of nuclei and nuclear morphology. Spermatogonia (sg) have dense, uniformly stained nuclei. They undergo mitotic transit amplification divisions to give rise to spermatocysts containing 64 nuclei in *Oncopeltus fasciatus* (Ewen‐Campen et al., 2013). Posterior to the spermatogonia undergoing transit amplification divisions, the spermatocytes (sc) undergo meiosis to form haploid spermatids (st), which then differentiate into spermatozoa (sz). (b) Higher magnification (12×) image of a representative testis tubule stained for both DNA (DAPI, cyan blue) and dividing cells (α‐pHH3 antibody; magenta). In this image, within the apical Region 1 (demarcated with the yellow dotted line) 5 spermatocysts containing synchronously dividing spermatogonia were labeled (yellow arrows). Occasionally, single nuclei were labeled with antibody (yellow star). These are likely to represent endoduplication of the cyst cell nuclei and were not included in the counts as they were clearly not spermatogonia or spermatocytes given that all nuclei within a cyst divide synchronously. Posterior to Region 1, in Region 2 (demarcated with the white dotted line), spermatocysts at the boundary of the spermatocytes and spermatid are labeled with α‐pHH3. While typically there are fewer spermatocysts dividing in this region, in this image there were 5 spermatocysts labeled as they progressed through meiosis, identified by the number of nuclei within the cyst (white arrows). Occasionally single cells were labeled (white star) in this region, again, presumably cyst cell nuclei

*O. fasciatus* is a hemipteran bug with a sequenced genome (Panfilio et al., 2019) that has been used as a physiological and developmental model system. Testis development (Economopoulos & Gordon, 1971) and the process of spermatogenesis (Ewen‐Campen et al., 2013; Schmidt et al., 2002) have been well documented. The testes of *N*. *femorata* and *O*. *fasciatus* have almost identical structures, consisting of seven testis tubules enclosed within a pigmented membrane. The organization along the axis of each testis tubule is also extremely similar, with stages in spermatogenesis from the apical end of the *N*. *femorata* testis tubule easily recognizable based on our understanding of the progression of spermatogenesis in *O*. *fasciatus* (Figure 1a). We examined rates of cell division within the spermatogonia. These transit amplification divisions produce multiple spermatogonial cells within a spermatocyst from the single diploid spermatogonial cell that arises from a germline stem cell. We compared the number of spermatogonial cysts showing evidence of cell division in adult males who were either autotomized or intact. Our prediction was that we should see evidence of an increase in the transit amplification divisions within autotomized males, indicating an increase in the level of sperm production.

## 2 METHODS

### 2.1 General husbandry

Adult *N*. *femorata* were collected from Starke, Florida (29.9803° N, 81.9848° W), and Live Oak, Florida (30.2642°N, 83.1768°W), between 30 May and 20 June 2019. The second generation of offspring from these wild‐caught bugs was raised in plastic cups (118 × 85 × 148 mm) with soil and a rooted *Opuntia mesacantha* ssp. *lata* cactus pad with ripe cactus fruits. Nymphs were kept at densities of 2–16 bugs per cup with temperatures between 24 and 28℃ and 60%–70% humidity. These cups were housed in a closed room under T5 HO fluorescent bulbs on a 14:10 L:D cycle and monitored every 48 hr to determine the date of 4th instar emergence. In February 2020, within 10 days of becoming 4th instars and being assigned to treatment groups, the bugs were transported to the Moore Lab at the University of Georgia, Athens, USA. The cups of *N*. *femorata* were secured in plastic trays and transported in the back of a covered truck bed. Upon arrival at the Moore Lab, the bugs were placed in similar rearing conditions as in the Miller Lab.

### 2.2 Experimental bugs

The impacts of autotomy at the onset of the penultimate stage on spermatogenesis were investigated as hemipterans are known to experience dramatic testes growth in these later stages of juvenile development (Economopoulos & Gordon, 1971). Once nymphs reached the 4th (penultimate) instar, they were randomly divided into one of two treatments: induced autotomy of the left hind limb or no autotomy (baseline control). Autotomy was induced by grasping the left hind limb by the base close to the body with forceps, allowing the bug to pull away and create a break at the joint between the trochanter and femur (Emberts et al., 2016). Once the treatments were applied, the 4th instar nymphs were placed on cactus in groups of 3–4 siblings per cup as conspecific density can impact development. Within high concentrations of *N*. *femorata*, faster developing siblings have much larger body sizes compared with the last siblings to develop into adults, which suggests strong competition between siblings (Allen & Miller, 2020). By placing nymphs in groups of 3–4, the impacts of conspecific density on development were minimized and consistent for the groups. Within 48 hr of becoming adults, the male bugs were separated into their own individual cups with a cactus pad and fruit.

### 2.3 Dissection and staining of testes

Between 21 and 28 days post‐adult emergence, the testes were removed from males. At this age, males are sexually mature. In *O*. *fasciatus*, most testis development occurs in juveniles, with some sperm maturation during sexual maturation (Economopoulos & Gordon, 1971). Once sexual maturation is reached, sperm production is at a “steady state” until mating. Thus, changes with age (days past adult emergence) in virgin males were not predicted for virgin sexually mature males. So for convenience, we grouped males at this stage as a single developmental stage, sexual maturation, rather than by age (days past adult emergence). Individual tubules were separated from the outer membrane for fixation and staining. Testis tubules from males within a treatment were pooled for staining if they were dissected on the same day. The testis tubules were fixed in 4% formaldehyde in PBS plus 0.1% Triton X‐100 (PBT) for 30 min. The tubules were stained with α‐phosphohistone H3 Ser10 (pHH3) primary antibody (Millipore antibody 06‐570, Sigma‐Aldrich). α‐pHH3 stains for chromosome condensation in preparation for mitosis and meiosis (Hans & Dimitrov, 2001; Prigent & Dimitrov, 2003). The secondary antibody was an Alexa Fluor goat anti‐rabbit 647 (Thermo Fisher Scientific). Following antibody staining, the tubules were stained with DAPI (0.5 μg/mL PBT) to visualize nucleic acids. Stained tubules were mounted in Mowiol 4–88 mounting medium (Sigma‐Aldrich). Slides were kept in boxes to limit light exposure and stored at 4℃ until visualized. The testis tubules were visualized with a Zeiss LSM 710 Confocal Microscope (Zeiss) at the UGA Biomedical Microscopy Core Facility. All testis tubules were photographed and included in the analysis.

### 2.4 Analysis of division rates within spermatogonia and spermatocytes

To test the prediction that autotomized males would show higher division rates, estimated from the number spermatocysts positively stained with α‐pHH3, than intact males, the number of spermatocysts stained with the α‐pHH3 antibody was scored in the photographs of individual testis tubules from males in the two treatments. The images were divided into the two regions, and stained spermatocysts were counted separately for Region 1, containing spermatogonia undergoing mitotic transit amplification divisions, and Region 2, containing spermatocytes undergoing meiotic division (Figure 1b). Only positively stained spermatocysts were scored. Results were reported on a single testis tubule basis. Single cells were occasionally stained, perhaps representing endoreplication within the cyst cells that enclose the spermatocysts (Figure 1b). These were not included in the analysis. Prior to analysis, the photographs were coded by an independent observer to allow for the data to be collected blind with respect to treatment. To check for interoperate error, two people counted a subset of images for stained spermatogonia in Region 1 and spermatocytes in Region 2. There was good agreement between the two sets of data indicating that the scoring was reliable. Values for positively stained spermatocysts ranged from 0 to 20 in fixed intervals. Visual inspection of the data indicated that the distributions met the assumptions of ANOVA. We analyzed 36 testis tubules for each treatment. We analyzed the data with a one‐way ANOVA with fixed effect using JMP Pro version 14. Power was calculated within the model function of JMP Pro v.14 with *α* = 0.05.

## 3 RESULTS

Regions of spermatogenesis were easily identifiable within the testis tubule (Figure 1a) based on our understanding of spermatogenesis in *O*. *fasciatus* testis tubules (Ewen‐Campen et al., 2013; Washington et al., 2020). At the apical tip of the testis tubule, there were spermatocysts containing spermatogonia. Spermatogonia divide mitotically and are recognizable by their relatively dense, uniformly stained nuclei. Posterior to the spermatogonia are the spermatocytes that undergo meiosis to form the haploid spermatids. Spermatids undergo differentiation to form spermatozoa.

**FIGURE 2:**
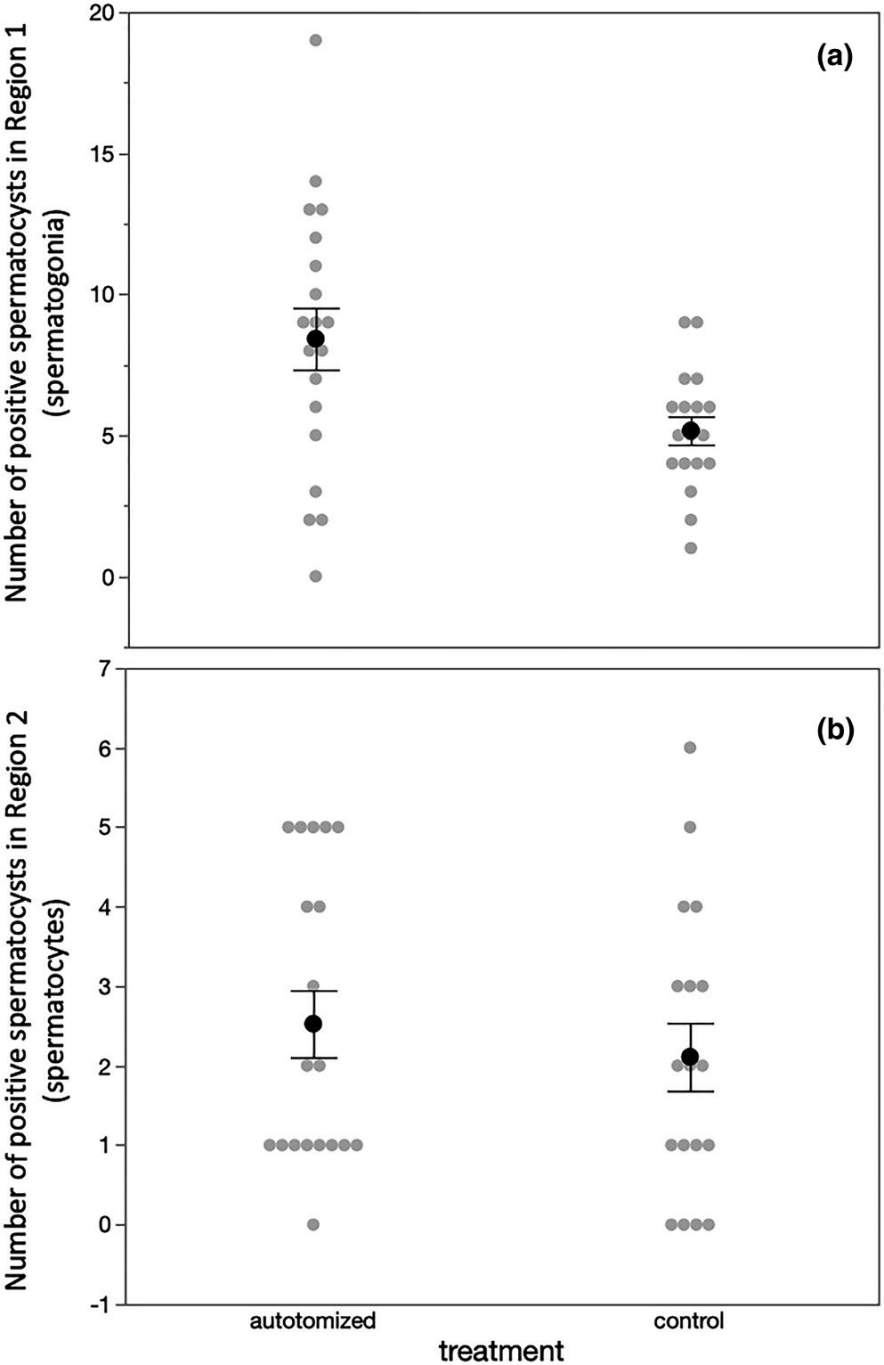
The number of spermatocysts within a single testis tubule from control males and autotomized males of the leaf‐footed cactus bug *Narnia* *femorata* that were positively stained with an α‐pHH3 antibody. (a) Spermatocysts in Region 1 containing spermatogonia from autotomized males were stained more frequently than those in the testis tubules of control males. (b) There was no difference in the number of spermatocysts in Region 2 containing spermatocytes undergoing meiosis between autotomized and control males. Black dots indicated the mean value for each treatment, and gray dots are individual data points. Each error bar is constructed using 1 standard error from the mean

Spermatocysts in the most apical region of the testis tubule (Figure 1b; sg) were more likely to be stained with α‐pHH3 antibody than spermatocysts in the region below (Figure 1; sc) where spermatocytes are undergoing meiosis (*F* = 34.723, *df* = 1, 63, *p* < 0.001), indicating that rates of cell division were greater in the spermatogonia than spermatocytes, as expected. Among spermatogonia undergoing transit amplification divisions, autotomized males had a higher number of spermatogonial spermatocysts stained with α‐pHH3 than control males (Figure 2a; *F* = 7.034, *df* = 1, 35, *p* = 0.012, power = 0.732). Among spermatocytes undergoing meiosis, there was no difference in the number of spermatocysts stained with α‐pHH3 (Figure 2b; *F* = 0.479, *df* = 1, 35, *p* = 0.494, power = 0.103).

## 4 DISCUSSION

While many studies have investigated the evolutionary outcomes of the trade‐off among pre‐ and postcopulatory strategies, fewer have investigated the mechanisms by which that trade‐off is mediated. We took advantage of the wealth of knowledge about a related model insect, *O*. *fasciatus*, to explore how loss of a weapon during testis development impacted spermatogenesis in *N*. *femorata*. We found an increase in testis size and fertilization advantage in autotomized male *N*. *femorata* that has been previously documented (Joseph et al., 2018; Miller et al., 2019; Miller et al., 2021) was associated with an increased rate of mitotic divisions in spermatogonia. Plasticity in sperm numbers and quality under variable conditions has been explored (Bunning et al., 2015; Dávila & Aron, 2017; Joseph et al., 2016; Moatt et al., 2014; Somjee et al., 2018), but few have examined the mechanism by which the increase in sperm numbers occurred. In *Drosophila melanogaster*, males respond to perceived sperm competition risk by increasing sperm production (Moatt et al., 2014). A recent study has shown that mating increases the division rate of germline stem cells in the testes of *D. melanogaster* through G protein signaling (Malpe et al., 2020). However, this sort of mechanistic studies depends on established molecular markers to identify the germline stem cells and niche cells, and genetic tools lacking in most species.

In *O*. *fasciatus*, and presumably *N*. *femorata*, sperm arise originally from germline stem cells at the tip of each testis tubule (Schmidt et al., 2002). As shown in *D. melanogaster* (Malpe et al., 2020), variation in sperm production could arise through the rate of production of germ cells through division of the germline stem cells. Alternatively, variation could arise through the modulation of the sperm production process (Extavour, 2013; Kaczmarczyk & Kopp, 2011; Moore, 2014; Ramm & Schärer, 2014). While we do not have the tools to examine germline stem cell turnover in either *N*. *femorata* or *O*. *fasciatus*, we have been able to show an impact of losing a weapon during larval development on the rate of spermatogonial divisions in adults.

The timing of autotomy in this study corresponded to a critical period of testis development. In *O*. *fasciatus*, testes are small and undeveloped in the 1st through 3rd instars. During the 4th instar stage of development, meiosis is initiated and spermatids begin to form (Economopoulos & Gordon, 1971; Schmidt et al., 2002). If, as we predict, the developmental timing is the same in *N*. *femorata*, then autotomy during the 4th instar stage of development is a time when the testis could benefit from increased resource allocation, either in increasing the number of germline stem cells (Kaczmarczyk & Kopp, 2011) or in increasing the number of spermatogonia that enter the maturation pipeline (Moore, 2014). The lack of difference in numbers of spermatocysts undergoing meiosis in Region 2 possibly could reflect the fact that these arose from spermatogonia born prior to autotomy.

The trade‐off between weapons critical for precopulatory mating success and traits critical for postcopulatory fertilization success has now been demonstrated in a number of species. Males that lack weapons tend to have increased fertilization success (Joseph et al., 2018; Somjee et al., 2018; Van den Beuken et al., 2019). Males that invest in precopulatory traits may not be able to fully invest in postcopulatory traits (Parker et al., 2013; Parker & Pizzari, 2010; Simmons et al., 2017). The constraint on investment in these strategies could be genetic (Filice & Long, 2018) (Reznick, 1985; Reznick et al., 2000) or depend on resource allocation (Joseph et al., 2018; Somjee et al., 2018). While there is much to be discovered about molecular and physiological control of spermatogenesis in *N*. *femorata*, studies such as this will allow researchers to dissect prospective targets of selection at a molecular level, opening up the potential for more targeted experimental manipulation. Ultimately, integrating the fitness outcomes of these trade‐offs with the molecular and cellular control mechanisms will allow us to examine the way in which selection shapes, or is constrained by, mechanism.

## CONFLICT OF INTEREST

The authors declare there are no conflicts of interest with this work.

## AUTHOR CONTRIBUTIONS

**Katelyn R. Cavender:** Conceptualization (equal); Writing‐original draft (lead). **Tessa A. Ricker:** Data curation (equal); Writing‐review & editing (equal). **Mackenzie O. Lyon:** Data curation (equal); Writing‐review & editing (equal). **Emily A. Shelby:** Data curation (equal); Writing‐review & editing (equal). **Christine W. Miller:** Conceptualization (equal); Writing‐review & editing (equal). **Patricia J. Moore:** Conceptualization (lead); Data curation (equal); Formal analysis (equal); Writing‐original draft (lead).

### OPEN RESEARCH BADGES

This article has earned an Open Data Badge for making publicly available the digitally‐shareable data necessary to reproduce the reported results. The data is available at https://datadryad.org/stash/dataset/doi:10.5061/dryad.5hqbzkh5m.

## Data Availability

The datasets analyzed for this study are made available by the authors through the publicly available Dryad Digital Repository (https://datadryad.org/stash/dataset/doi:10.5061/dryad.5hqbzkh5m).
